# Thyroid Nodules Detected by Contrast-Enhanced Magnetic Resonance Angiography: Prevalence and Clinical Significance

**DOI:** 10.1371/journal.pone.0149811

**Published:** 2016-02-26

**Authors:** Hyun Kyung Lim, Sung Tae Park, Hongil Ha, Seo-youn Choi

**Affiliations:** 1 Department of Radiology, Section of Neuroradiology, Soonchunhyang University Seoul Hospital, Soonchunhyang University College of Medicine, Seoul, South Korea; 2 Department of Radiology, Hallym University Medical Center, Hallym University Sacred Heart Hospital, Anyang, South Korea; 3 Department of Radiology, Section of Neuroradiology, Soonchunhyang University Bucheon Hospital, Soonchunhyang University College of Medicine, Bucheon, South Korea; H. Lee Moffitt Cancer Center & Research Institute, UNITED STATES

## Abstract

**Background and Purpose:**

Incidental thyroid lesions are frequently found on contrast-enhanced magnetic resonance (CE-MR) angiography. The purpose of this study is to determine the prevalence of thyroid incidentalomas detected by CE-MR angiography and to evaluate their clinical significance by correlation with ultrasound (US) and cytopathological results.

**Materials and Methods:**

We retrospectively reviewed 3,299 consecutive CE-MR angiography examinations performed at our institution between January 2010 and March 2013. Two radiologists evaluated the CE-MR angiography imaging in consensus regarding the presence, location, and vascularity of thyroid incidentaloma. We correlated these findings with follow-up US and cytopathologic results.

**Results:**

The prevalence of thyroid incidentalomas detected by CE-MR angiography was 4.6% (152/3,299 patients). CE-MR angiography showed hypervascularity in 86.8% (145/167), isovascularity in 8.4% (14/167), and hypovascularity in 4.8% (8/167) of thyroid nodules compared to vascularity of thyroid parenchyma. Among the patients with thyroid incidentaloma, 34 patients (22.4%) were followed by US examination, and all 36 nodules on CE-MR angiography were detected on follow-up US. Of these nodules, 9 (25%) nodules were classified as probably benign, 26 (72.2%) as indeterminate, and 1 (2.8%) as suspicious malignant nodule. Among the 16 nodules with available cytopathologic results, 12 nodules were benign, 2 nodules were follicular neoplasm, and 2 nodules showed non-diagnostic results.

**Conclusion:**

Incidental thyroid nodules were found in 4.6% of CE-MR angiography examinations. Because the high incidence of indeterminate US feature among thyroid incidentaloma, when a thyroid incidentaloma is detected on CE-MR angiography, further evaluation with US should be performed.

## Introduction

The detection of thyroid nodules is growing, and thyroid nodules are frequently detected on ultrasound or other imaging modalities such as computed tomography (CT), position emission tomography (PET), and magnetic resonance imaging (MRI). Definition of thyroid incidentaloma is a thyroid nodule detected on imaging modalities performed for the purpose other than the detection of thyroid nodule, and many thyroid nodules are identified incidentally. The prevalence of thyroid incidentaloma on imaging modalities other than ultrasound (US) and its clinical significance have been reported by several earlier studies [1–8].

The prevalence of thyroid incidentalomas shows a steady growth with the increased use of various imaging modalities, such as US, CT, and MRI [9]. However, there are few studies that have reported the prevalence of incidental thyroid nodules on CT or MRI, and its clinical significance. Youserm et al. [10] reported that the prevalence of thyroid incidentalomas was 15.6% among 123 CT scans and 108 MRI examinations of the head and neck. A more recent study [5] reported the prevalence of 16.8% among 734 neck CT examinations.

MR angiography is widely used as a routine examination for the evaluation of neck vessels, and we have frequently noted focal hyperenhancing lesion within the thyroid gland in daily practice. However, to our knowledge, there have been only 2 reports on the prevalence and the radiologic-cytopathologic correlation for thyroid incidentaloma found on MR angiography [1, 6], and each study evaluated 624 and 2010 MR angiographies, respectively.

Therefore, the purpose of our study was to evaluate the prevalence of thyroid incidentaloma found on MR angiography and to evaluate the clinical significance of thyroid incidentaloma detected by MR angiography by correlation with US findings and cytology results in a larger study population.

## Materials and Methods

This retrospective study was approved by Soonchunhyang University Seoul Hospital Institutional Review Board for human investigation, and written procedural consent was obtained from all patients prior to MR angiography, US and US-guided fine-needle aspiration biopsy (FNAB).

### Patients

Between January 2010 and March 2013, 3299 consecutive patients underwent CE-MR angiography examinations using the 1.5 T MR scanner at out institution. All these MR examinations were reviewed to evaluate the presence of visible thyroid nodules. Two patients underwent MR angiography twice during that period, and the first MR angiography was used for evaluation. There was no any change observed in follow-up MRA in these two patients. The subjects consisted of 1989 men and 1310 women, and the mean ± SD age was 67 ± 13 years (range, 27–91 years). All examinations were performed as a part of health screening, for risk evaluation regarding cerebrovascular stroke, or for the diagnosis and follow-up of acute cerebrovascular stroke.

### Contrast-Enhanced MR angiography technique

All MR angiography was performed with the 1.5 T MRI system (Sonata, Siemens HealthCare, Erlangen Germany) using a 8-channel neurovascular array coil (Siemens HealthCare, Erlangen Germany). The parameters were as follows: Field of view, 39 cm; TR/TE, 4.3/1.31 ms; flip angle, 30°; matrix, 202 x 384; number of average, 1; section thickness, 1mm; no interslice gap. A total of 72 slices were acquired and the acquisition time was 17 seconds. A bolus of 15 ml of gadodiamide at a concentration of 0.5 mmol/ml (Omniscan, Nycomed, Princeton, NJ) was injected at 2 ml/s with a flush of 15 ml of saline at the same rate, using a power injector (Medras, Medrad, Indianola, PA). Two phases of pre-contrast and arterial phase were obtained. And, only the arterial phase images were displayed on picture archiving and communication system (PACS), using 3D maximum-intensity-projection (MIP) reconstruction.

### US and US-guided FNAB procedure

All US examinations and US-guided FNABs were performed by 1 of 2 radiologists (H.K.L. and S.T.B.) with 8 and 18 years of experience, respectively, in performing thyroid US examination and FNAB. All US examinations were performed using iU 22 (Philips Medical Systems, Bothell, WA, USA) equipped with linear high-frequency probe (5–14 MHz). FNABs were performed using a free-hand technique with a 21 gauge needle and a 10-ml plastic syringe by the same radiologist who performed the US exam. FNAB was performed according to the indication suggested by Korean Society of Thyroid Radiology [11].

### Image Analysis of MR angiography and US

Two radiologists (H.K.L., S.C.), who were blinded to the US finding and cytology results, retrospectively evaluated the CE-MR angiography imaging in consensus, regarding the presence, location, and vascularity of thyroid nodules. The location of thyroid nodule was divided as right lobe, isthmus, and left lobe for the anatomical correlation of the thyroid nodule between CE-MR angiography and US. Each lobe was divided into 3 locations including upper, middle and lower portion. The vascularity of a thyroid nodule was categorized as hypervascular, isovascular, or hypovascular compared to the vascularity of thyroid parenchyma.

US images were reviewed in the patient with thyroid incidentaloma detected on MR angiography. The thyroid incidentalomas on MR angiography were consensually matched to the corresponding nodules on US by 2 radiologist (H.K.L., H.H), who were unaware of the cytopathologic findings. US findings for the nodules were evaluated for the following features: location, size (longest diameter) and US assessment. US features of thyroid nodules were already documented in the reports according to the recommendation of the Korean Society of Thyroid Radiology [11]. Final US assessments were categorized as suspicious for malignancy, indeterminate, or probably benign. Findings of suspicious for malignancy category were defined as having at least one of the following features: a taller-than-wide shape, a spiculated margin, markedly hypoechoic, microcalcification or macrocalcifications. Probably benign findings were defined by the presence of a spongiform nodule, cystic or predominantly cystic nodules with a comet-tail artifact. Indeterminate findings were defined as all US findings other than those indicating probably benign nodule or suspicious malignant nodules [12, 13]. FNA cytology diagnoses were classified into 6 categories according to the Bethesda System [14].

### Laboratory Examination and Clinical Follow-up

Laboratory examinations including thyroid function test and thyroid antibody test were recorded. In the patients without follow-up US examination, Lab-test within 7 days with MR angiography was recorded. In the patients with follow-up US examination, Lab-test performed between MR angiography and US was recorded. If available, thyroid scan result was also recorded.

We also searched electronic medical records from the time point of patient enrollment to December 2015.

## Results

Total 167 thyroid incidentalomas were detected in 152 patients (59 male, 93 female; mean ± SD age, 68 ± 12 years; range, 30–87 years) among the 3299 patients who had MR angiography. The prevalence of thyroid incidentaloma detected by CE-MR angiography was 4.6% (152/3299 patients). All patients with thyroid incidentaloma had no history of thyroid hormone supplementation. One thyroid nodule was detected in 140 patients, 2 nodules in 10 patients, 3 nodules in 1 patient, and 4 nodules in 1 patient. 55 nodules were located in right lobe, 3 nodules in isthmus, and 109 nodules were located in left lobe.

The vascularity of thyroid incidentalomas were hypervascular in 145 nodules (86.8%), isovascular in 14 nodules (8.4%), and hypovascular in 8 nodules (4.8%).

**Fig 1:**
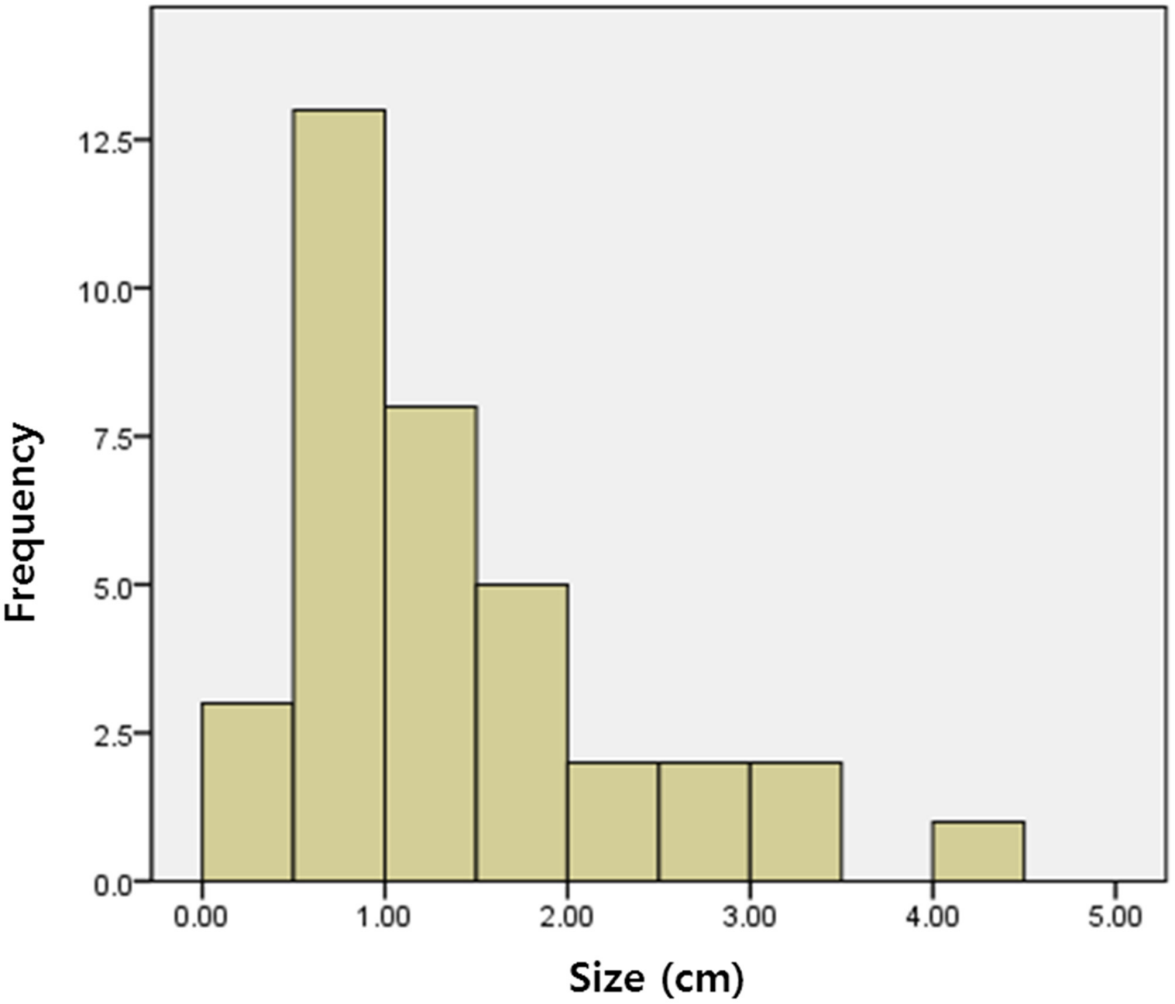
Size distribution of thyroid nodule detected on US examination.

**Fig 2:**
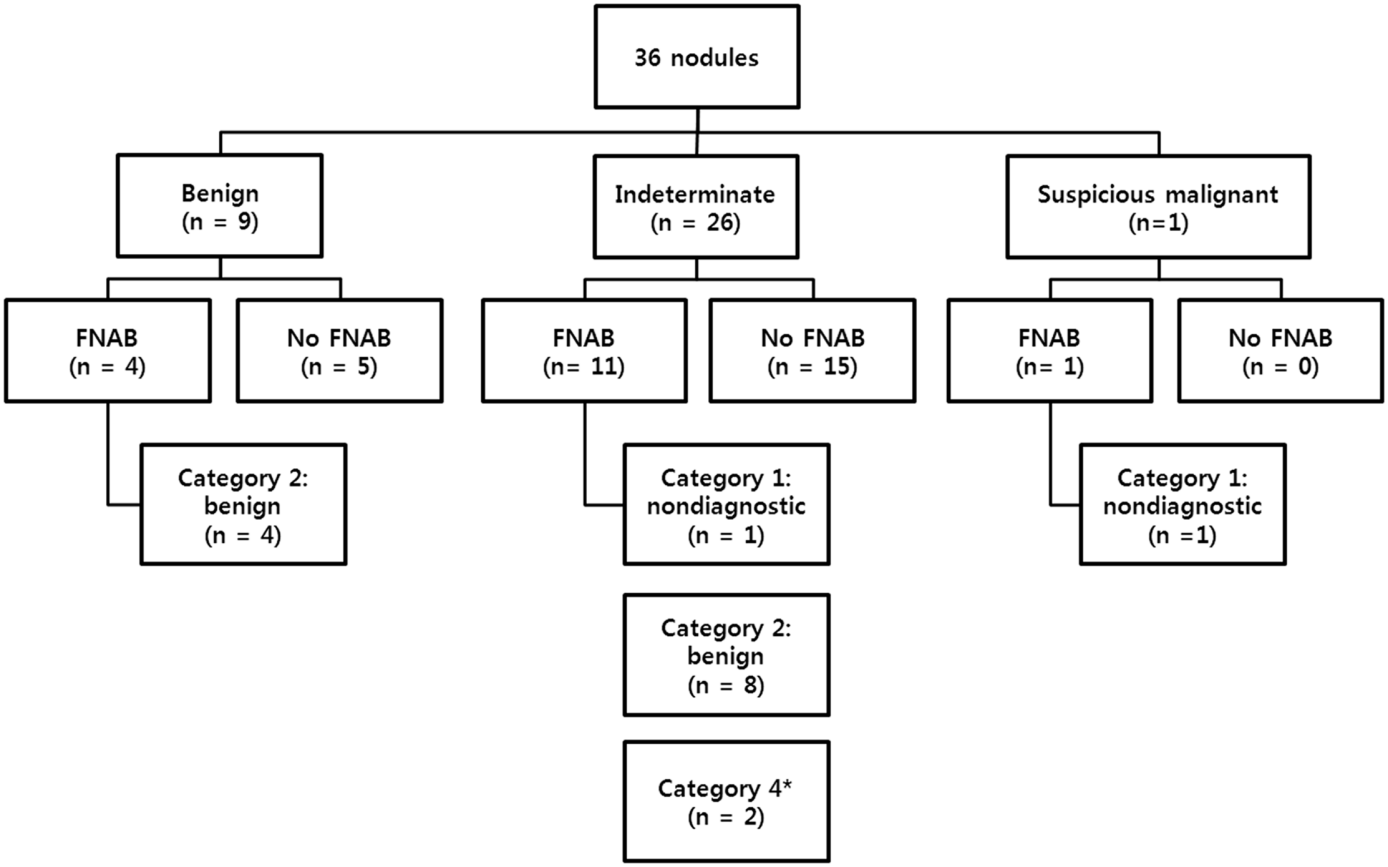
US assessment and US-guided FNAB results of 36 thyroid incidentalomas on MR angiography, followed by US. * Follicular neoplasm Hurthle cell type (n = 1) and suspicious for follicular neoplasm (n = 1).

Among the 152 patients with thyroid incidentaloma, 34 patients (22.4%) were followed by US examination, and all 36 nodules (one nodule in 32 patients, two nodules in 2 patients) on CE-MR angiography were detected on follow-up US. The time interval between CE-MR angiography and US was 82 ± 109 days. The size distribution of thyroid nodule on US was presented in Fig 1. The mean largest diameter of thyroid nodules was 1.35 ± 0.89 cm (range, 0.29–4.07 cm). Of these 36 nodules, 9 (25%) nodules were classified as probably benign, 26 (72.2%) as indeterminate, and 1 (2.8%) as suspicious malignant nodule on US examination (Fig 2).

**Fig 3:**
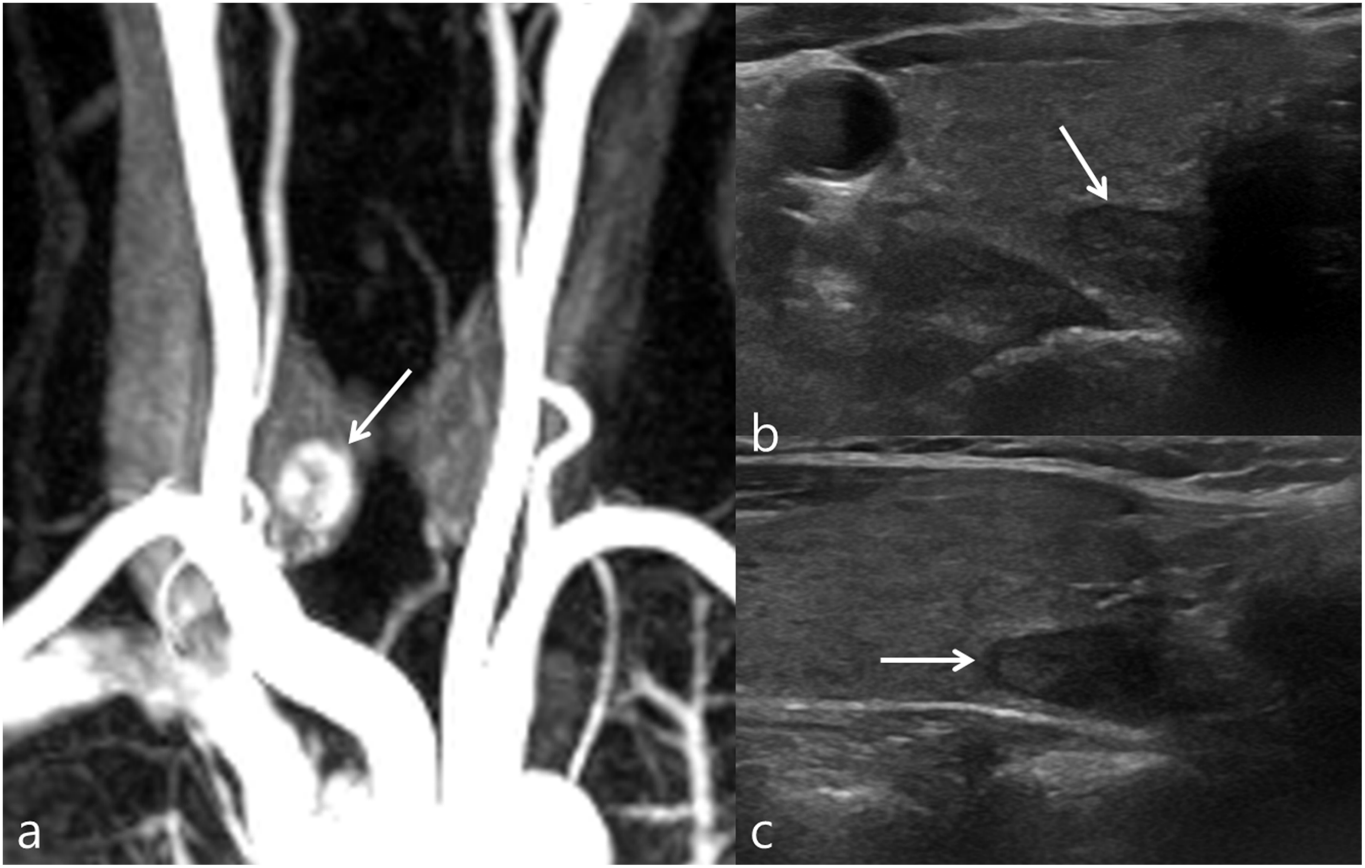
Incidental hypervascular thyroid nodule in a 72-year-old female. (a) CE-MR angiography shows hypervascular thyroid nodule (arrow) in the right lobe. (b) Transverse US images reveal 1.15 cm size, well-defined hypoechogenic solid nodule with smooth margin and ovoid shape (arrows). This nodule was classified as indeterminate nodule.

**Fig 4:**
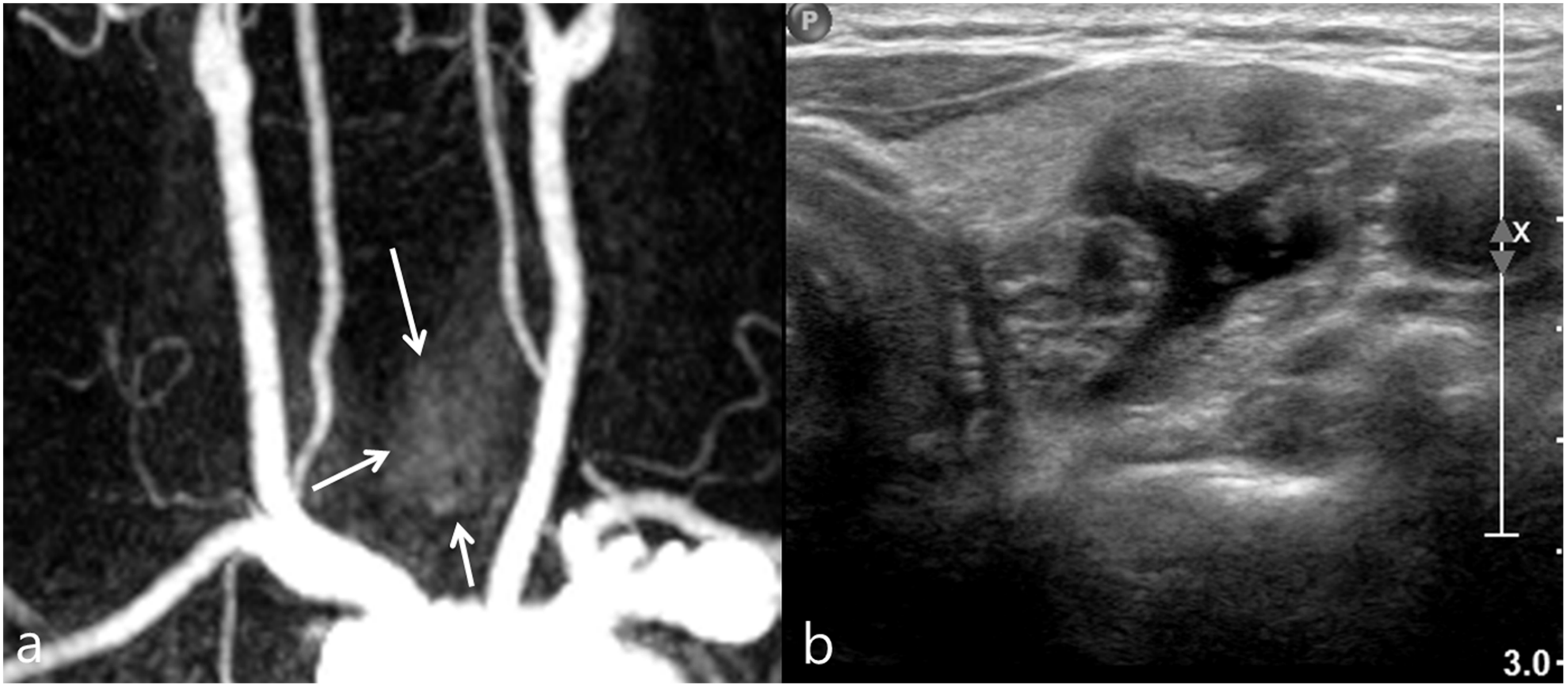
Incidental isovascular thyroid nodule in a 53-year-old female, subsequently diagnosed as benign follicular lesion by US-guided FNAB. (a) CE-MR angiography shows ill-defined thyroid nodule (arrows) with similar vascularity compared to the thyroid parenchyma. (b) Transverse US image of left lobe reveals 2.15 cm size, predominantly solid nodule with spongiform appearance. This nodule was classified as probably benign nodule.

**Fig 5:**
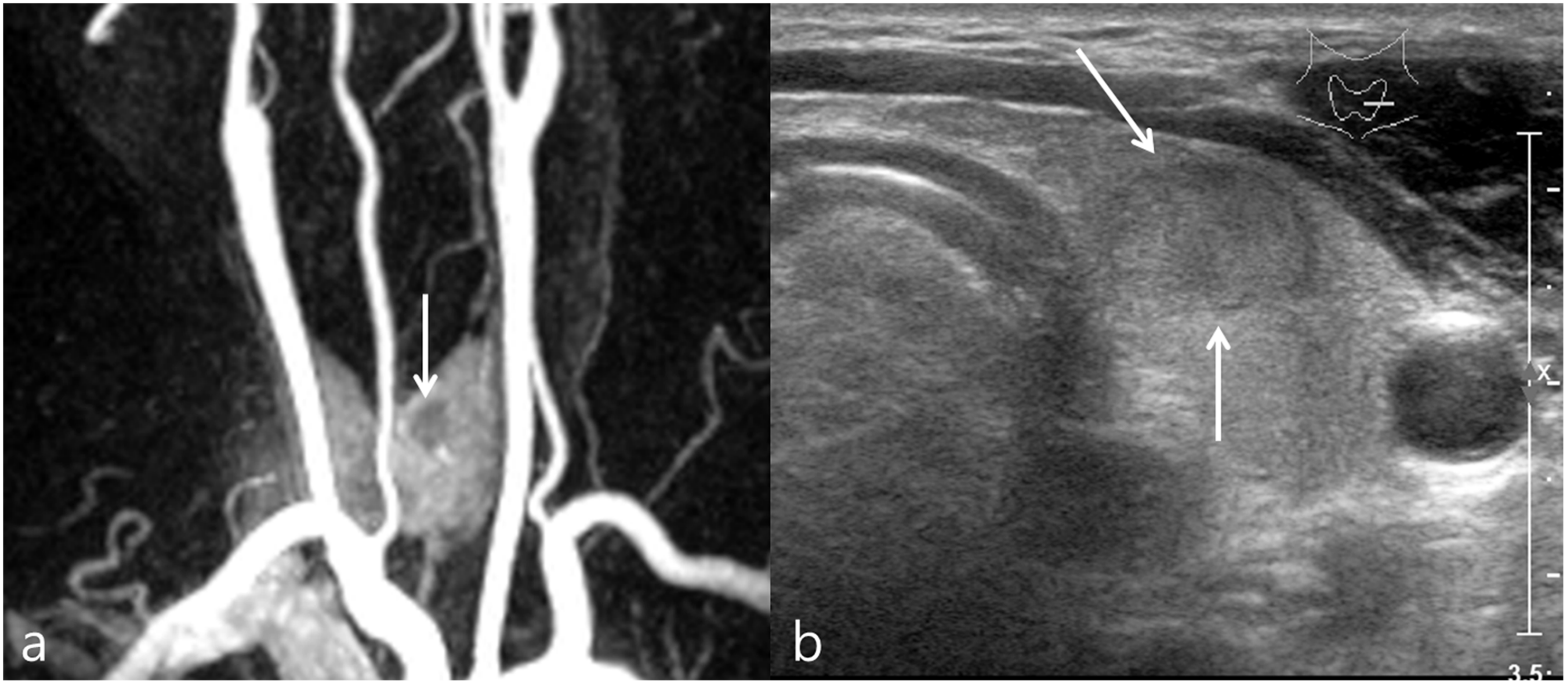
Incidental hypovascular thyroid nodule in a 61-year-old man, subsequently diagnosed as Hashimoto’s thyroiditis by US-guided FNAB. (a) CE-MR angiography shows left thyroid nodule (arrow) with homogenous hypovascularity. (b) Transverse US image of left lobe reveals 1.38 cm size, isoechogenic solid nodule (arrows). This nodule was classified as indeterminate nodule.

US-guided FNAB was done in 16 nodules of the 36 nodules identified on US. The US assessment of thyroid incidentalomas detected on CE-MR angiography, and FNAB results were summarized in Fig 2. US-guided FNAB was performed according to the indication that was recommended by KSThR [11]. According to the guideline, indeterminate thyroid nodule more than 1 cm is the indication of FNAB. According to the FNAB indication, 11 indeterminate nodules were followed by US-guided FNAB. 15 indeterminate nodules did not proceed to FNAB due to 1) denial of FNAB for old age (at least 72-years old) and other severe medical problem such as cerebrovascular accident or malignancy (5 nodules), 2) transfer to other hospital (3 nodules), and 3) no FNAB indication due to small size (7 nodules). Among 7 indeterminate nodules that did not meet the FNAB indication, two nodules were followed by US 1 year after, and showed no change. Other 5 nodules were not followed by US due to old age (at least 72-years old) and other severe medical disease such as cerebrovascular accident or malignancy. Two patients with Bethesda category 4 (follicular neoplasm or suspicious for a follicular neoplasm) underwent operation, and one nodule was confirmed as follicular adenoma, and another nodule was confirmed as nodular hyperplasia. Among the 2 nodules with non-diagnostic results, one nodule showed suspicious malignant features on US and was confirmed as nodular hyperplasia after surgery. Another nodule with non-diagnostic result and indeterminate US feature was not evaluated further. Figs 3–5 represented typical incidentalomas with hypervascular, isovascular and hypovascularity, each.

Among 118 patients with thyroid nodule on MRA, but without US follow-up, 27 patients were not followed due to transfer to other hospital or unknown cause. 5 patients died for cerebrovascular accident or non-thyroidal malignancy. There was no patient who later presented with thyroid cancer.

**Fig 6:**
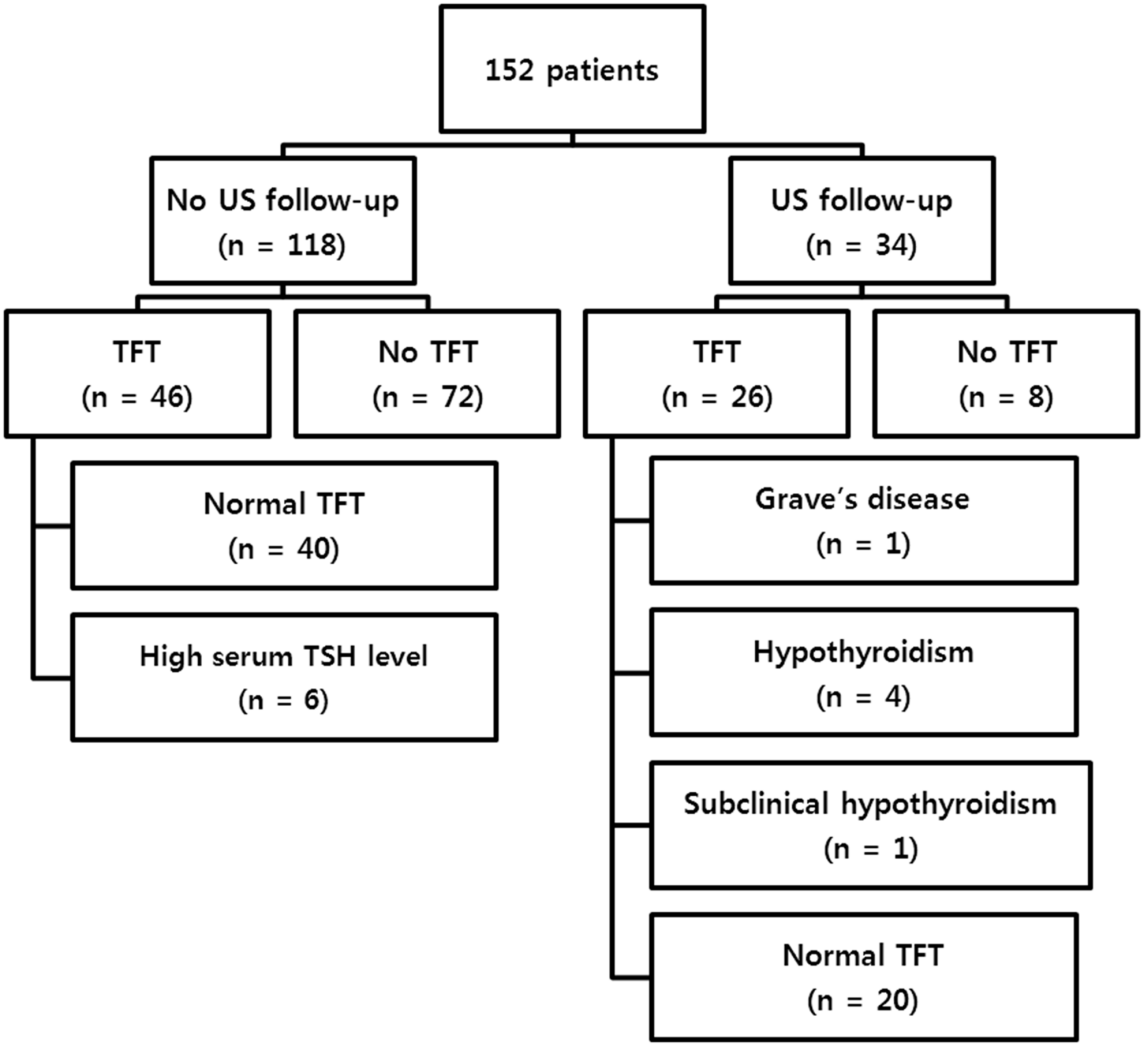
Summary of thyroid function test and thyroid antibody test results in 152 patients with incidental thyroid nodule on MR angiography.

Thyroid function test and thyroid antibody test results of all enrolled patients were summarized in the Fig 6. In the patients without available thyroid function test, there was no medical record of thyroid disease other than nodule. Among 118 patients without US follow-up, 6 patients showed high serum TSH level. But, follow-up was loss due to transfer to other hospital. Among 26 patients with US follow-up and thyroid function test, 6 patients show abnormal Lab-result suggesting Grave’s disease, hypothyroidism, subclinical hypothyroidism.

Only one patient with Grave’s disease underwent thyroid scan (Tc-99m) and increased uptake (7.2%) and one cold nodule was found. Because this nodule did not meet the FNAB indication, FNAB was not followed.

## Discussion

We found that the prevalence of incidental thyroid nodules on CE-MR angiography was 4.6%, and most of them revealed indeterminate features on US.

The prevalence of thyroid incidentaloma on MR angiography in our study was lower than that of previous studies, which reported 7.8% and 5%, each [1, 6]. The lower prevalence in our study may have been due to different MR technique and different patient population. Whereas previous studies used dynamic CE-MR angiography technique, our study was done with static CE-MR angiography technique. Dynamic CE-MR angiography has hemodynamic information on the vessels and thyroid gland. Therefore, there is a greater chance to detect thyroid nodules at multiple time points [1, 6]. However, the static technique has been more widely used as CE-MR angiography than the dynamic technique, and our study evaluated the largest population (3299 patients) to date. Therefore, the prevalence of thyroid incidentaloma in our study may be more accurate than previous studies.

The conspicuity of thyroid incidentaloma on MR angiography resulted from hyperenhancement or decreased enhancement compared with the adjacent thyroid parenchyma. Additionally, the degree of enhancement represented the degree of vascularity. In our study, hypervascularity exceeding that of adjacent thyroid parenchyma was the most common pattern among incidental thyroid nodules detected on MR angiography, occurring in 86.8% of such lesions. This result was concordant with results of previous studies [1, 6]. However, hypervascularity of incidentaloma was nonspecific. Because of small number of US follow-up cases, analysis about relationship between nodule vascularity on MRA and FNA result was not possible. When incidental thyroid lesions are incidentally detected on MR angiography, further evaluation using the US exam should be performed, irrespective of the pattern of enhancement. Because the risk of malignancy according to the US feature is well known, the next step in the diagnosis depends heavily on the US feature. Furthermore, among the 36 nodules with US examination, 72.2% showed indeterminate features. The thyroid nodule with indeterminate feature on US is usually recommended for further evaluation by US-guided FNAB according to the size [9, 11]. Because the high prevalence of indeterminate nodule on US in our study, identification of thyroid nodule on CE-MR angiography should necessitate further evaluation with FNAB.

We assessed only one nodule as suspicious malignant feature among 36 nodules, which were evaluated by US examination. Furthermore, no malignant tumor was confirmed by pathology. Previous studies also reported a low prevalence of malignancy. Lohan et al. reported that 1 of 12 cytologically confirmed nodules was malignant, and Choi et al. reported that 1 of 16 cytologically confirmed nodules was malignant [1, 6]. However, the true prevalence of malignancy of thyroid incidentalomas found on MR angiography could not be determined accurately because only a small fraction of incidentalomas were histologically confirmed in previous studies: 1.9% in the study by Lohan et al., and 0.8% (17/2010) in the study by Choi et al.

It was reported that incidental thyroid nodule on MRA should be followed by US by Choi et al. and Lohan et al. However, in our institution, the radiologist who read MR angiography frequently ignored thyroid nodule on MRA, and did not recommend thyroid US examination. He reported thyroid nodule and recommended thyroid US only in the case of conspicuous thyroid nodule that was strong hypervascular or large in size on MRA (18 patients). However, these 18 patients did not undergo thyroid US due to transfer to other hospital (3 patients), poor medical condition such as cerebrovascular accident or other malignancy (7 patients), very old age over 80 year-old (2 patients), and uncertain cause (6 patients). In patient with advanced malignancy or very old age, physicians thought that thyroid US or US-guided FNAB did not affect the prognosis of the patients. All thyroid US examinations in 34 patients were conducted for patient’s need (28) or abnormal thyroid function test result (6). Therefore, only small number of thyroid nodules was followed by US.

There are several limitations to our study. First, US examination was done in a fraction of cases. This was due to the retrospective design of our study. Second, a limited number of cytological comparisons were performed. Therefore, verification of the true prevalence of malignancy was not possible. A well-designed prospective study including pathologic confirm will be needed for further evaluation of the true clinical significance of incidental thyroid nodules detected on MR angiography.

## Conclusion

In conclusion, thyroid incidentalomas were found in 4.6% of CE-MR angiography examinations. Because the high incidence of indeterminate US feature among thyroid incidentaloma, when the thyroid incidentaloma is detected on CE-MR angiography, further evaluation with US should be performed.

## Supporting Information

S1 File Whole data set of our study. This exel file is the data of 152 patients with thyroid incidentaloma detected on MR angiography.
(XLSX)

## Abbreviations

CE-MR: contrast-enhanced magnetic resonance
US: ultrasound
CT: computed tomography
MRI: magnetic resonance imaging
PET: position emission tomography
FNAB: fine-needle aspiration biopsy
